# Understanding Age-Related Longitudinal Dynamics in Abundance and Diversity of Dominant Culturable Gut Lactic Acid Bacteria in Pastured Goats

**DOI:** 10.3390/ani13162669

**Published:** 2023-08-19

**Authors:** Doaa E. ElHadedy, Chyer Kim, Adnan B. Yousuf, Zhenping Wang, Eunice N. Ndegwa

**Affiliations:** 1 Agricultural Research Station, Virginia State University, Petersburg, VA 23806, USA; doaa.elhadedy@temple.edu (D.E.E.); ckim@vsu.edu (C.K.); ayousuf@vsu.edu (A.B.Y.); zwang@vsu.edu (Z.W.); 2 National Centre for Radiation Research and Technology NCRRT, Radiation Microbiology Department, Egyptian Atomic Energy Authority (EAEA), Cairo 11787, Egypt

**Keywords:** lactic acid bacteria, goats, fecal samples, RAPD-PCR, 16S sequencing, diversity

## Abstract

**Simple Summary:**

Evaluating age-based differences in the resident lactic acid bacteria in the gut of healthy animals is important for potential probiotic species identification and development. This gives an understanding of unique bacterial populations that are well-adapted for survival in each age group. In goats, the resident gut lactic acid bacteria diversity in different age groups has not been fully described. The objective of the study was to evaluate the abundance and identity of the lactic acid bacteria populations in different age groups of goats from birth until ten months age. We detected higher counts of lactic acid bacteria in young goat kids before weaning than goats after weaning. Additionally, we detected different lactic acid bacteria populations in the different age groups evaluated. The findings give insight on potential lactic acid bacteria species that could be targeted for the development of probiotics for different ages of goats. These results may also broadly imply that when selecting potential bacteria for probiotic evaluation in other host animals, it is important to consider the target age to ensure success of establishment in the gut.

**Abstract:**

Understanding gut lactic acid bacteria (LAB) in healthy hosts is an important first step in selecting potential probiotic species. To understand the dynamics of LAB in healthy goats, a cohort of thirty-seven healthy new-born goat kids was studied over a ten-month period. Total LAB was quantified using SYBR green qPCR. Seven hundred LAB isolates were characterized using microscopy, M13 RAPD genotyping and 16S rDNA sequencing. The highest and lowest LAB counts were detected at one week and ten months of age, respectively. Diverse LAB species were detected, whose identity and prevalence varied with age. The main isolates belonged to *Limosilactobacillus reuteri*, *Limosilactibacillus fermentum*, *Lactobacillus johnsonni*, *Ligilactobacillus murinus*, *Ligilactobacillus salivarius*, *Limosilactobacillus mucosae*, *Lactiplantibacillus plantarum*, *Ligilactobacillus agilis*, *Lactobacillus acidophilus/amyolovolus*, *Pediococcus* spp. and *Enterococcus* spp. Uniquely, *L. reuteri* and *Pediococcus* spp. were most common in pre- and peri-weaned goats, while *Lactobacillus mucosae* and *Enterococcus* spp. were predominant in goats one month and older. Based on RAPD genotyping, *L. reuteri* had the highest genotypic diversity, with age being a factor on the genotypes detected. This data may be relevant in the selection of age-specific probiotics for goats. The findings may also have broader implications by highlighting age as a factor for consideration in probiotic bacteria selection in other animal hosts.

## 1. Introduction

Lactic acid bacteria (LAB) include Gram-positive, catalase-negative, aero-tolerant anaerobes, acid-tolerant, organo-trophic, non-motile, fermentative cocci or rod-shaped bacteria that produce lactic acid as an end product to carbohydrate metabolism [1,2]. They are found in a wide range of nutrient-rich ecological niches including food products, fruits and in the cavities of animals [2,3,4,5]. The bacteria genera in this group include *Lactobacilli*, *Pediococci*, *Lactococci*, *Enterococci*, *Streptococci* and *Leuconostoc* [2,6]. LAB found in the gastrointestinal tract of animals play a key role in enhancing immunity and maintaining gut homeostasis in animals [7,8].

Many species of LAB are considered probiotics and defined as live microorganisms that are generally regarded as safe (GRAS), which, when given to a host in the right amounts, exert a beneficial effect [9]. The most widely studied and characterized LAB belong to the genus *Lactobacillus* due to their well-characterized potential for use as probiotics [4]. In animals and humans, other important LAB species applied as probiotics include the *Pediococcus*, *Enterococcus*, *Streptococcus* and *Lactococcus* species. For the development of probiotics, LAB isolates from healthy host target species are recommended for evaluation for potential benefits [10]. This ensures success of establishment in the gut of host animal and in the potential realization of health benefits [11,12]. Consequently, understanding the resident LAB species in healthy animals becomes a prerequisite in developing species-specific probiotics. Various techniques are used to study previously undescribed microbial populations including isolation, microscopy, biochemical characterization and molecular methods [13]. High throughput sequencing has offered great insight recently as a tool to determine broad microbial diversity in many ecological niches including the gut of an animal. Several studies have recently utilized this tool to understand the dynamics of gut microbiota specifically in goats [14,15,16], including the differences in healthy and diseased animals [17] and the effect of probiotic supplementation [18]. For example, in the study by Wang and colleagues, they evaluated goat kids and their nursing does, age-based colonization shifts and several bacterial biomarkers that were detected in goats from birth to 56 days of birth [17]. These were subsequently correlated to volatile fatty acid concentrations. In sheep, specific bacterial populations were also associated with divergent feed conversion ratios [19]. Thus, using high throughput sequencing tools, specific bacterial populations can be mapped to desirable host performance and consequently targeted for isolation. As we gain these broad gut microbiome insights, traditional culture methods will continue to be complementary in further studying the identified microbial populations [20,21,22,23] and their mechanism of action. The availability of whole genome sequencing technology will also allow scientists to fully characterize isolates of further interest, including identifying probiotic-associated genes [24]. This will be important if the ultimate goal is to identify culturable populations for further technological applications and for improving the health and performance of animals.

The random amplified polymorphic DNA (RAPD) is a commonly applied, fast and inexpensive molecular tool for typing different types of bacteria in food, plant, animal and environmental microbiology [25,26,27,28,29,30]. Several studies have in the past reported success in using RAPD-PCR for the differentiation of large numbers of bacterial isolates including LAB strains [31,32,33,34]. Sequencing of conserved and variable regions of isolates followed by basic local alignment sequence tools (BLAST) offers great insight into the identification and diversity of new lactic acid bacteria [35]. In goats, the resident gut LAB diversity in different age groups has not been fully described. For potential probiotic species identification and development, evaluating age-based differences in the resident LAB in the gut of healthy animals will give an understanding of unique bacterial populations that are well adapted for survival in each age group. In this study, we used microbial isolation, microscopy and molecular tools to understand and describe the changes in the abundance, diversity and identity of the dominant culturable LAB microflora in the developing gut of a cohort of healthy pastured goats over a ten-month growing period.

## 2. Materials and Methods

### 2.1. Animals and Husbandry

The study was carried out at Virginia State University (VSU), USA in a small ruminant research unit. The goat flock comprised Myotonic and Spanish breeds bred in November, and kidding occurs in March/April every year. The goat kids are routinely born naturally on pasture and remain with their nursing does until weaning at approximately three months. Thereafter, the goats remain on pasture with daily supplementation, with a corn–soybean ration at 2% body weight. Baled hay is supplemented as needed in winter. The pasture is mainly composed of eastern gammagrass (*Tripsacum dactyloides*) pasture with volunteer common Bermuda grass (*Cynodon dactylon*) and herbaceous annual grass legumes. A cohort of thirty-seven newborn goat kids were recruited into the study and followed until ten months of age. Fecal samples were first collected within 48 h (2 days) of birth, followed by seven days (7), fourteen days (14), twenty-eight days (28) and fifty-six days (56) of age, day of weaning (0 DPW), 1 day after weaning (1 DPW), one week after weaning (7 DPW), one month after weaning (1 MPW), four months after weaning (4 MPW) and finally eight months after weaning (8 MPW). Animals were cared for according to an approved Virginia State University Institutional Animal Care and Use Protocol (VSU AACUC #2018-001).

### 2.2. Fecal Sample Collection

At each sampling, individual fecal samples were collected from the rectum either using a sterile swab moistened in one mL (1 mL) of phosphate-buffered saline (PBS) (2 days, 7 days and 14 days) or rectally, with a lubricated gloved finger (all other samplings). Samples in swabs were vigorously vortexed before 200 µL was removed for the enrichment step (2.3). Fecal samples were subsequently transported in ice to the laboratory for microbial isolation.

### 2.3. Microbial Isolation and Identification of Lactic Acid Bacteria in Fecal Samples

An in-house initial enrichment and isolation protocol followed by standard anaerobic LAB incubation and isolation protocols were followed. On the day of sampling, fecal samples were subjected to an initial 48 h enrichment in de Man Rogosa Sharpe (MRS) broth containing Tween 80 (Sigma-Aldrich, St. Louis, MO, USA) under anaerobic incubation using Mitsubishi AnaeroPack^®^-(MGC, Tokyo, Japan) Anaero sachets in jars. Two hundred milligrams (200 mg) of solid fecal sample or 200 µL of fecal solution was added to 3 mL of MRS broth media. For samples collected using a swab, the fecal solution was retrieved by vigorously vortexing the swab. The enrichment was stored in 20% glycerol at −80 °C until further analysis. Isolation and identification of lactic acid bacteria from glycerol-preserved MRS enrichment were carried out according to a standard procedure. Briefly, under aseptic condition transfer, 20 µL of the enrichment was transferred to 3 mL of MRS broth media and incubated under the anaerobic condition at 37 °C for 48 h. Serial dilutions of the resulting growth were prepared up to 10^6^ and 20 µL from each dilution plated on MRS agar (MRSA) (Sigma-Aldrich) followed by incubation under the anaerobic condition at 37 °C for 48 h. Plates with well-separated single colonies were identified. Representative colonies showing different morphologies on the plates were picked. Colonies were evaluated under a light microscope using Methylene blue dye 8% under oil immersion and at 100×. Colonies were transferred to 2 mL of MRS and incubated anaerobically for 48 h. Isolates in broth were preserved in 20% glycerol at −80 °C until further analysis.

### 2.4. Total DNA Extraction

Total DNA was extracted from the MRS enrichment for quantification of LAB using a simple boiling method. For this method, two (2 mls) of the MRS enrichment broth were transferred to 2 mL tubes. This was followed by centrifugation at 10,000× *g* rpm for 4 min to pellet the bacteria. The pellet was washed twice with 1 mL of molecular-grade water (Corning^®^, Corning, NY, USA), with each washing step followed by centrifugation and the discarding of the supernatant. After the final washing step, 100 µL of molecular-grade water was added to the pellet, vortexed and heated at 100 °C for 10 min. The suspension was centrifuged again at maximum speed for 4 min, and the resulting supernatant containing DNA was removed. Concentration and purity of DNA was measured using NanoDrop™ (Themofisher Scientific, Waltham, MA, USA) spectrophotometer and stored at −20 °C or −80 °C until use. Only samples with a purity of 1.8 and above were used for further processing in PCR protocols.

### 2.5. LAB Abundance Quantification Using qPCR

Quantification of LAB was conducted with SYBR green qPCR protocol using the 16S ribosomal RNA gene primers of lactic acid bacteria as previously described [36] (see Supplementary Table S2 for all primers used in the study). Extracted DNA was diluted to between 10 and 100 ng of DNA. A standard curve was generated using PCR products from amplification of *Lactobacillus acidophilus* ATCC 4356 DNA and primers F: 5-AGCAGTAGGGAATCTTCCA-3 and R5, 5-CACCGCTACACATGGAG-3. The samples were run in duplicate, and a cycle threshold (Cq) was used to calculate and quantify the log count of LAB in each sample using the standard curve generated. A negative control sample containing water was included in each assay run. Evaluation of the melting curve was used to detect and confirm amplification of LAB spp. in each sample. The limit of detection of the lactic acid bacteria was 10 genome copies. For all bacteria detection and quantification, the amplification protocol followed the Applied Biosystems PowerUp ^TM^ SYBR Green Master Mix reaction set up recommendations except for the annealing and extension temperature that were unique for the primer pair used in this study. The total reaction volume was 10 µL for all reactions. The qPCR program was forty cycles comprising annealing temperature of 58 °C for 15 s, extension at 72 °C for 30 s and final extension at 80 °C for 30 s with data collection at 80 °C, followed by a melting curve analysis cycle.

### 2.6. Microbial DNA Extraction, RAPD-PCR and Analysis of RAPD-PCR Fingerprints

A commercial microbial extraction kit, Bactozol^®^ kit (MRC, Cincinnati, OH, USA), was used to extract individual LAB genomic DNA for the RAPD-PCR following manufacturer’s protocol. The final DNA pellet was reconstituted in molecular-grade water. To ensure dissolution of the DNA pellet, a final heating step at 55 °C for 5 min followed by centrifugation was carried out. The supernatant was transferred to a fresh DNase/RNase-free microcentrifuge tube. DNA concentration and purity were measured using Nanodrop, and samples with a ratio of 260/280 not less than 1.8 were used as a template for RAPD genetic fingerprinting using the M13 primer (5-GAGGGTGGCGGTTCT-3) [37]. The PCR amplification reaction was carried out following the parameters described in [38]. The protocol includes one cycle of 10 min at 95 °C for initial denaturation, 40 cycles of 1 min at 95 °C (denaturation), 20 s at 42 °C (annealing) and 2 min at 72 °C (elongation), as well as a final extension of 10 min at 72 °C. PCR amplification was performed in a SimpliAmp thermal cycler (Applied Biosystems, Waltham, MA, USA) in a 25 μL reaction mixture containing the following: 12.5 µL of master mix (AmpliTaq Gold™ 360 Master Mix), 2 µL of primer M13 (20 picomoles), DNA (250 ng) and molecular-grade water. A total of 20 µL of each PCR product was loaded into the wells of a 1.5% agarose gel, electrophoresed at 120 V for 90 min and stained with ethidium bromide. A 1 kb DNA ladder (NEB) was used as a DNA molecular weight marker. The gel was visualized with gel documentation system E-Gel Imager (Life Technologies, Carlsbad, CA, USA), and images were saved for further analysis. The images of the RAPD gels were captured using gel documentation system software that comes with the E-Gel Imager. The images were saved as jpeg files, and analysis was conducted manually. The number and size of bands on the RAPD-PCR fingerprint were counted in comparison to DNA standards (1 kb ladder). The banding pattern obtained from the gel for each isolate was scored in a binary data format. The scoring was based on the presence or absence of the band. A score of zero indicated the absence of the band, whereas one indicated the presence of a particular band. A dendrogram that draws the distance calculation for the different band profiles was carried out using an unweighted pair-group method analysis UPGMA [39], and cluster analysis was carried out using the Past software 4.03 [40].

### 2.7. PCR Amplification and DNA Sequencing of Lactic Acid Bacteria

Many isolates shared the same M13 RAPD banding profiles. Consequently, select isolates processed for sequencing represented all the unique profiles detected with M13 RAPD typing. The universal primers employed for sequencing targeted a 676 bp fragment of the 16S rDNA gene, primer 9f (5-GAGTTTGATCCTGGCTCAGGA-3) for positions 9–30 (LC1 from and primer 685r (5-TCTACGCATTTCACCGCTAC-3) for positions 666–685) (according to the Escherichia coli numbering system) [41]. Each 25 µL reaction mixture contained 12.5 µL of master mix (AmpliTaq Gold™ 360 Master Mix), 1 µL of forward and reverse primer. A total of 200 ng of DNA from each isolate was used as a template DNA. The reactions were carried out in SimpliAmp thermal cycler (Applied Biosystems, USA) with the following parameters: a 10 min initial denaturation at 95 °C followed by 40 cycles of 1 min at 95 °C, 40 s at 55 °C and 90 s at 72 °C, and the final extension step for 10 min at 72 °C. The PCR products were electrophoresed at 120 V on a 1% agarose for 60 min. The 676 bp PCR product was purified using E.Z.N.A^®^ pure cycle purification kit (Omega Bio-Tek Inc., Norcross, GA, USA). NanoDrop™ spectrophotometer was used to check on DNA concentration and purity before submission for sequencing. The sequencing of the products was performed at Eton Bioscience Inc., Research Triangle Park, NC, USA. Sequence chromatogram analysis was evaluated for quality and chimeras using Eton Biosciences free online sequence chromatogram software located on their site. Sequences of good quality were uploaded in the NCBI basic local alignment search tool (BLAST) (www.ncbi.nlm.nih.gov/BLAST (accessed on 17 June 2023)) for alignment of the 16S rDNA sequences to evaluate the highest similarity (>99%) with other known lactic acid bacteria spp. in the gene bank.

### 2.8. Statistical Analysis

Abundance of LAB data was recorded in log genomes per 100 ng of DNA for each sampling point. Data between sampling points were compared using repeated ANOVA and Tukey HSD test. Frequency of detection of individual LAB isolates in goats was compiled using descriptive statistics for each age group evaluated. Proportions of animals harboring each of the LAB species were calculated. Comparison of proportions was carried using the online Medcalc’s comparison of proportion software tool (https://www.medcalc.org/calc/ (accessed on 17 June 2023)).

## 3. Results

### 3.1. Changes in Abundance of LAB during Growing Period

**Figure 1:**
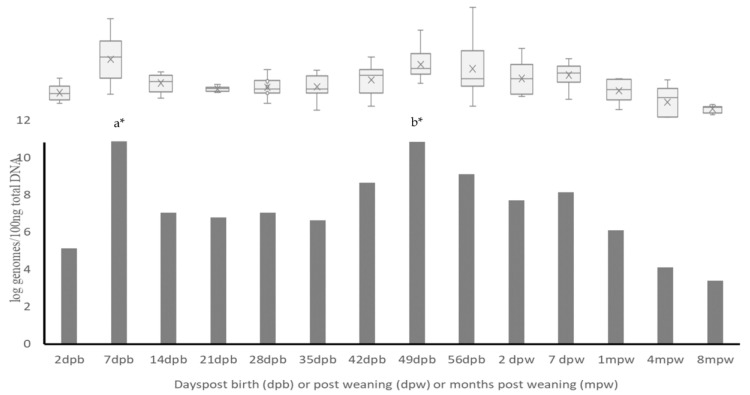
Changes in total LAB (*Lactobacillus* and *Pediococcus* spp.) counts and individual animal total count variability in the gut of growing pastured goats (dpb—days post birth, dpw—days post weaning and mpw—months post weaning). a*—LAB counts on the sampling point were significantly higher (*p* < 0.05) than the lowest seven sampling points; b*—LAB counts on the sampling point were significantly (*p* < 0.05) higher than the lowest six sampling points.

The qPCR utilized in this study is designed to quantify *Lactobacillus* and *Pediococcus* spp. Overall, *Lactobacillus* and *Pediococcus* spp. were detectable using qPCR in all the goat kids as early as two days of birth and throughout the study period (Figure 1). In general, higher counts of LAB were detected during the pre-weaning period, and counts declined gradually after weaning, with lowest counts being detected eight months after weaning. The counts ranged from a high of 11 log genomes/100 ng of DNA detected at one week of age to a low of 3.4 log genomes/100 ng of DNA detected at eight months post weaning. Two peaks of high counts of LAB were detected, one at one week of age and another at seven weeks of age. There was a significant increase in the total counts detected from two days of age to one week of age when the highest counts were detected (*p* < 0.05). This was followed by a significant decrease (*p* < 0.05) at two weeks of age that was maintained until 49 days of age when the LAB counts peaked again. Variability in individual animal LAB counts was also detected at different sampling points. The highest individual animal variability was detected at seven and fifty-six days of age, while the lowest variability was detected at three weeks and eight months of age.

### 3.2. Diversity and Prevalence of LAB at Different Age Groups of Goats

A diverse species of lactic acid bacteria was detected in the goats evaluated in this study in all ages evaluated (Table 1). Four hundred and forty LAB isolates (440) from goats ranging from two days to ten months of age were selected based on microscopy and uniqueness of RAPD profiles and were sent for sequencing (see Supplementary Figure S1 for typical RAPD gel). All isolates described here could be matched with other related LAB in the NCBI GenBank with BLAST at or above 99% similarity. Based on sequencing and BLAST results, LAB distribution in this study belonged to the following LAB groups: *L. reuteri*, *L. fermentum*, *L. johnsonni*, *L. ingluviei*, *L. murinus*, *L. salivarius*, *L. plantarum*, *L. acidophilus/amyolovoulus*, *L. brevis*, *L. oris*, *L. agilis*, *L.mucosae*, *L. animalis*, *L. saemnari*, *W. confusa*, *Pediococcus* spp. *(Pediococcus acidilactici, Pediococcus lolii* and *Pediococcus pentosaceus)* and *Enterococcus* spp. (Table 1). These appeared as rods of different sizes (*Lactobacillus* spp.), or coccobacilli (*Pediococcus* spp.) and cocci (*Enterococcus* spp.), as identified with a light microscope (Supplementary Table S1).

At each sampling point, there was individual animal variability in the identity and the number of unique LAB populations detected. The highest number of goats having more than three different LAB species detected at one time was on the first day of sampling (0 DPB) where 30% of the goat kids harbored three (3) different LAB species at one time, including some goat kids that harbored as many as four (4) different species of LAB simultaneously. Throughout the study over 50% of the goats had at least two different species of LAB at all sampling points evaluated.

**Figure 2:**
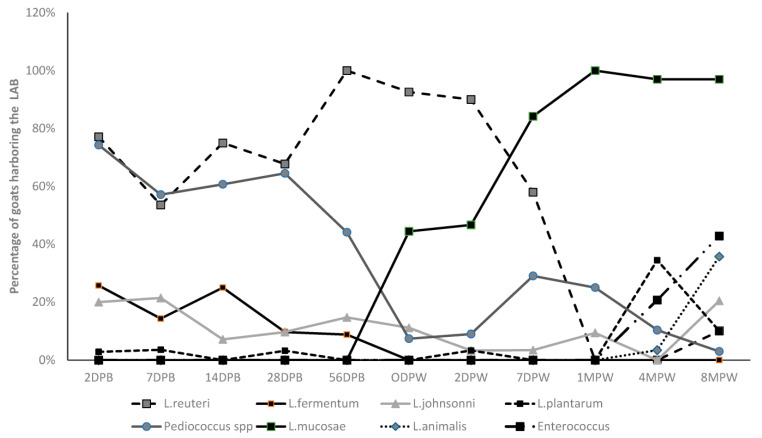
Changes in dominant LAB species in different age groups of pastured goats (DPB—days post birth, DPW—days post weaning and MPW—months post weaning).

There were significant differences in the prevalence and identity of the predominant LAB populations detected between pre-weaned/peri-weaned goats and animals over one month of age (Figure 2). While *Lactobacillus* and *Pediococcus* species were detected in all age groups of goats evaluated in this study, *Enterococcus* spp. were exclusively cultured from goats older than four months (Figure 2 and Table 1).

In this study, *L. reuteri* was the most common lactic acid bacteria detected in pre-weaned and peri-weaned goats (up to 7 days after weaning), followed by *P. acidilactici* (Figure 2 and Table 1). During the pre- and peri-weaning period, the number of goat kids harboring *L. reuteri* was over 60% at each sampling point, which was significantly higher (*p* < 0.05) than all the other LAB species detected. On the other hand, at one month after weaning (1 MPW) and beyond, *L. reuteri* was not detected in any animal at 1 MPW and 4 MPW, and only three goats harbored this species at eight months after weaning (8 MPW). The second most abundant species of LAB in the pre- and peri-weaned group of goats was the *Pediococcus* species (Figure 2 and Table 1). In all pre-weaned goat kid age groups except at 56 days of age, *Pediococcus* species were detectable in over 50% of the animals, which was also significantly higher (*p* < 0.05) than the other *Lactobacillus* species (excluding *L. reuteri*) detected. The frequency of isolation of this species similar to *L. reuteri* declined significantly (*p* < 0.05) after 56 days of age. In animals at 4 MPW and 8 MPW, *Pediococcus* spp. was detected in two goats at each sampling point. Both *L. fermentum* and *L. johnsonni* were also detected in at least 10% number of goats at each sampling point during the pre-weaning period. Specifically, *Lactobacillus fermentum* was only detected in pre-weaned goats and in none beyond sampling points after 56 days of age. *L. johnsonni* was detected in animals after weaning (less than 10 percent) at 8 MPW when it was detected in 21% of goats.

On the other hand, *L. mucosae* was the predominant LAB detected in goats during the post-weaned period (Figure 2 and Table 1). This species was first detected at the day of weaning (ODPW), (44%), which significantly (*p* = 0.002) increased to over 80% at 7 DPW. Subsequently, the species was detected in 100% of all the animals sampled at 1 MPW, 4 MPW and 8 MPW. Another LAB species predominantly detected in 4 MPW and 8 MPW goat was *L. animalis.*

The other LAB detected including, *L. murinus*, *L. plantarum*, *L. brevis*, *L. salivarius* and *L. acidophilus*, were rare and sporadic but were detected in all age groups evaluated, while *L. agilis* was detected on the day of weaning (ODPW) and one week after weaning (7 DPW). A number of other LAB detected in this study including *L. saerimneri and W. confus/cibaria* were rare and only detected in goats older than one month (Figure 2 and Table 1).

Lactic acid bacteria belonging to the *Enterococcus* species were exclusively detected in post-weaned animals at four months post weaning (4 MPW) and 8 MPW. At 4 MPW, 21% of animals harbored this species, and the proportion of animals increased significantly (*p* = 0.07) to 43% at 8 MPW.

### 3.3. Frequency of Detection of LAB Strains in Individual Animals Following Repeated Sampling

Data on the frequency of detection of LAB in individual animals over time may give insight on the competitive ability of each species in the gut of the animals over time. We evaluated the frequency of detection of predominant LAB in the same animal in subsequent samplings over the study period. *L. reuteri* and *Pediococcus* spp. were the most consistently detected in subsequent samplings in individual animals during the pre-weaning period. Uniquely, for most animals, once *L. reuteri* was detected at 2 days of age (77%), it was also detected at least two other times in subsequent samplings in the same animal in over 50% of the animals until 7 days after weaning. Additionally, for animals where this strain was not detected on the first sampling day, (23%), it was detected in 100% of the animals in subsequent samplings. This may indicate the stability of this *L. reuteri* species in goats during this growing period. The other stable LAB species detected in this age group of goat kids was *Pediococcus* spp. This species was also detected at least two other times in subsequent samplings in over 50% of goat kids once detected at two days of age until 56 days of age. *L. johnsonni* was detected inconsistently in goats with 20% of goat kids at two days of age. Of these, 5% had this strain of LAB detected one week later, and none were detectable at one month of age (28 days). At 56 days of age, 15% of the goat kids had *L. johnsonni* detected, including two animals that had originally harbored this strain at 2 days and three others that did not have it at 2 days of age. This species was detected sporadically in other animals throughout the sampling period, including in goats at 8 MPW. Similarly, there was no clear indication of stability in the establishment of *L. fermentum* in goats, although it was only detected in the pre-weaned goats. In eight (22%) of the animals that harbored *L. fermentum*, at 2 days of age, it was only detected again in two of these animals in subsequent samplings—one at one week of age and the other at 56 days of age. The strain was also detected once in eight other goat kids in subsequent samplings, but these were different animals at each point. *L. johnsonni* was also detected in goats at 1 MPW and 8 MPW, but these were different animals at each sampling point.

The stability of *L. mucosae* in older goats close to and after weaning was evident. By 1 MPW, 100% of the goats harbored and continued to harbor this species during the next two samplings. The *Enterococcus* species was first detected in goats at four months post weaning (4 MPW), where six animals had this species. However, at 8 MPW, more animals harbored this species than those detected at 4 MPW, although not the same animals that had been detected at 4 MPW.

At one week of age, one animal harbored *L. plantarum*, then three at two weeks of age, and this strain was detected in only one of the goat kids in two subsequent samplings. In post-weaned goats, *L. plantarum* was detected at 1 DPW, 4 MPW and 8 MPW, but no consistent isolation from same animal was evident. The other LAB strains, including *L. murinus*, *L. ingluviae*, *L. brevis*, *L. agilis*, *L. oris*, *L. saerimneri*, *L. animalis*, *L. salivarius* and *L. acidophilarus/amylovorus*, were detected sporadically in individual animals.

### 3.4. Genotypic Diversity of LAB from Goats as Evaluated with RAPD

**Figure 3:**
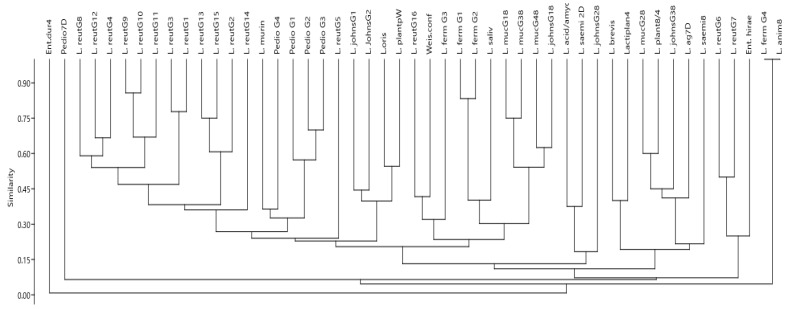
Dendrogram showing similarity index of 49 representative LAB M13 RAPD genotypes isolated from goats; Lreut = *L. reuteri*, Ljohns = *L. johnsonni*, Lferm = *L. fermentum*, LplantPW = *L. plantarum* (pre-weaned), Lmurin = *L. murinus*, Lacid/am = *L. acidophilus/amyolovorous*, Lbrevis = *L. brevis*, Loris = *L. oris*, Lsaliv = *L. salivarius*, Pedio = *Pediococcus* spp., Weisconf = *W. confusa*, Lmuc = *L. mucosae*, L.plant8/4 = *L. plantarum* isolated from goats at 4 MPW and 8 MPW, Lsaemi = *L. saemneri*, Lanim = *L. animalis*, Lag7D = *L. agilis* isolated at 7 DPW, Ent. hirae = *Enterococcus hirae*, Ent dur = *Enterococcus durans*, *Lplant4* = *L. plantarum* (isolated at 4 MPW) and *L. Johns (G18*, *G28* and *G38) = L. johnsonni* genotypes isolated from goats at 8 MPW.

Genotyping was conducted using M-13 RAPD-PCR, which had shown high reproducibility and repeatability for both LAB and other bacterial species in other studies [31,35,42,43]. Several RAPD genotype patterns that differed interspecies and intra-species were detected, indicating a diverse number of strains or sub-strains of the LAB species in the goats in this study. The M13 primer generated between 1 and 25 unique bands in the LAB isolates evaluated a total of 49 different unique RAPD genotype patterns that were detected in this study, representing the different species of LAB detected. Out of these, 41 genotype patterns belonged to the *Lactobacillus* species, while 5 belonged to the *Pediococcus* spp., 2 belonged to *Enterococcus* spp. and 1 belonged to *W. confusa* (Figure 3).

**Figure 4:**
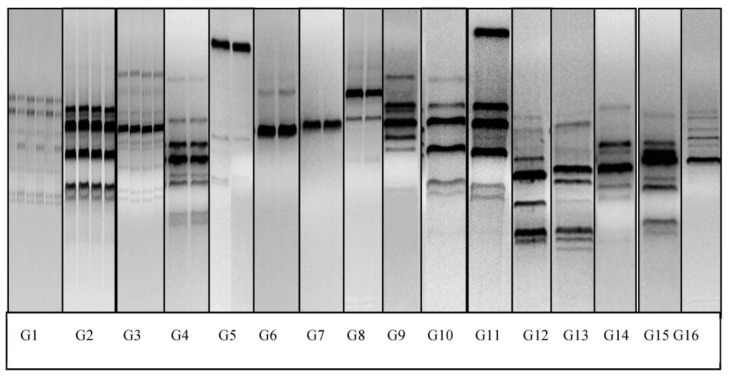
Representative M13 RAPD banding patterns of 16 *L. reuteri* genotypes from goats.

Among the *Lactobacillus* spp., *L. reuteri* showed the highest RAPD genotype diversity. In total, *L. reuteri* belonged to 16 different RAPD patterns (Figure 3 and Figure 4). Genotypes 1, 2 and 3 were the most commonly detected, representing over 60% of the isolates in the group. We detected a predominance of specific *L. reuteri* RAPD genotype patterns in specific age groups, while other patterns were detected across all the ages until weaning. Goat kids at 56 days of birth harbored the highest genotypic diversity of *L. reuteri*. In particular, *L. reuteri* isolates belonging to genotype 3 were exclusively detected in goat kids at the age of 56 days. Over 80% of the *L. reuteri* with genotype group 1 were detected in goat kids at the age of 56 days, while the rest were detected in goats at 28 days of age. Similarly, strains possessing genotype pattern 8 were detected in 28- and 56-day-old goat kids only. None of the *L. reuteri* genotype groups 1, 3 and 8 were detected in goat kids at 7 or 14 days of age. On the other hand, strains possessing the RAPD genotype 2 were detected in all ages evaluated in this study. Genotype 4 was detected in goats at 2 and 56 days of age, with genotype 5 detected at 2 and 28 days of age, genotype 6 at 2, 14, 28 and 56 days of age and genotype 7 at 2, 14 and 56 days of age. Some RAPD genotype patterns were more frequently detected in the study of animals than others were. 

**Figure 5:**
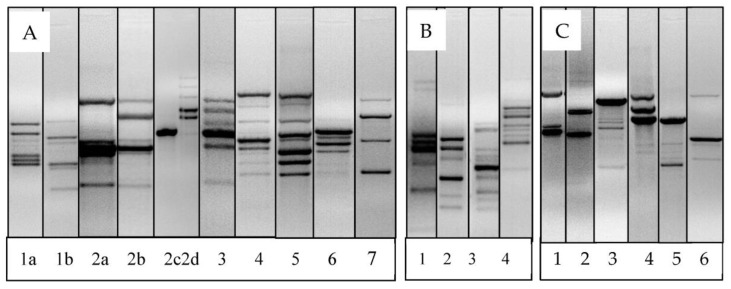
(**A**): Representative M13 RAPD patterns for *L. johnsonni* (1a and 1b), *L. fermentum* (2a, 2b, 2c and 2d), *L. murinus* (3), *L. plantarum* (4), *L. oris* (5), *L. acidophilus* (6) and *L. brevis* (7). (**B**): Representative M13 RAPD patterns for *Pediococcus* spp. (**C**): Representative M13 RAPD pattern for *L. mucosae* (1), *L. agilis* (2), *L. saerimneri* (3), *L. plantarum* (4) (4 MPW isolates) and *L. johnsonni* (5 and 6) (8 MPW isolates).

Overall, *L. reuteri* RAPD pattern 1, 2 and 3 were the most frequently detected in the study animals. RAPD genotypes 4, 5, 6 and 7 were detected in four to seven animals, while RAPD genotypes 8–17 were rare and detected in three or less animals during the study. Among all the different *L. reuteri* genotypes, there was overall a low similarity index (<10%) based on RAPD genotypes, although some genotypes were more similar than others. Among the three major genotypes (1, 2 and 3), the similarity index was 30%, but genotypes 1 and 3 had higher similarity index (78%). Other genotypes with over 75% similarity index included 9 and 10, also 13 and 15 (Figure 3 and 4). Six different genotypes of *L. johnsonni* were detected in different age groups of goats based on the RAPD patterns. Two of these were detected in pre-weaned animals, while in older animals at 8 MPW, four (4) unique genotypes were also detected (Figure 3). A RAPD gel representation of four of the genotypes detected in both pre-weaned and post-weaned goats is shown in Figure 5A (genotypes 1a and 1b) and Figure 5C (genotypes 6 and 7), respectively. The two genotypes detected in pre-weaned goats had about a 45% similarity index, while the three detected in older goats had less than 20% similarity. Most of the isolates (18) belonged to one major cluster (genotype 1a), while the other cluster had only 3 isolates (genotype 1b). Interestingly, isolates with genotype 1b were only detected in goats at 56 days of birth. Similarly, *L. fermentum* also had high clonality and formed one major cluster with (12) isolates (genotype 2a in Figure 5A), another cluster with 2 isolates (2b) and two other single genotype unique isolates (2c and 2d). The most common genotype (2a) was found across all pre-weaned age groups sampled, while genotype 2b was detected in goats at one week of age. The other two unique genotypes (2c and 2d) were detected in goats at 2 days of age. Most isolates of *L. murinus* (genotype 3) formed one cluster that had over a 95% similarity index. They were detected in goats at 28 and 56 days of age and one week after weaning (7 DPW). Other rarely detected Lactobacillus species isolated in different age groups of goats presented unique RAPD genotypes shown in Figure 5A. These include *L. salivarius*, *L. plantarum* (genotype 4 at 7 days of age), *L. oris* (genotype 5 at 28 days of age), *L. acidophilus/amylovorus* (genotype 6 at 56 days of age) and *L. brevis* (genotype 7 at 7 days of age). The *L. plantarum* and *L. oris* isolates had a similarity index of 60% and were closer to *L. johnsonni* than other *Lactobacillus* species.

There was little RAPD genotype diversity detected in *Pediococcus* spp. using the M13 primer (Figure 4). Most isolates formed one single cluster (genotype 1), while three other isolates presented unique genotype patterns (genotypes 2, 3 and 4). Figure 5B shows the M13 RAPD genotype patterns for the *Pediococcus* species.

The M13 RAPD pattern for all *L. mucosae* (Figure 5C-1) showed 100% similarity in banding pattern irrespective of the age of animal from which they were isolated. A similar clonality was detected for all *Enterococcus* spp. isolated. The RAPD banding pattern for *L. agilis*, *L. saerimneri*, *W. confusa*, *L. plantarum* (adult animal isolates) and *L. animalis* were all unique (Figure 5C; 2–6).

## 4. Discussion

Lactic acid bacteria in the gut of animals are major components of the microbiome that significantly contribute to the overall immune development and maintenance of gut homeostasis. In addition, lactic acid bacteria from healthy animals have been a source of potential bacteria for development as host specific probiotics or potentially other broad applications in food safety. Thus, understanding of LAB in healthy hosts becomes a prerequisite in developing host-specific probiotics. Consequently, the goal of our study was to describe the dynamics of the dominant culturable LAB species in healthy growing pastured goats. In this study, we report the abundance and diversity of LAB isolated from growing healthy pastured goats from two days of age to eight months after weaning using a quantitative PCR, microbial isolation, RAPD genotyping and partial 16S sequencing. The latter two techniques were able to offer insight into the diversity of LAB in goats, highlighting differences in identity and genotypes common in each age group. Anaerobic enrichment in MRS broth and plating on MRS agar yielded diverse species of LAB. Additionally, the M13 primer was successful in amplifying and discriminating the isolates described in the study, including differentiating genotypes of the same species.

The gut of pastured goats in this study was enriched with different species of LAB, whose abundance and diversity changed over time. Based on SYBR green molecular quantification, LAB was detected throughout the study period in goats, but the abundance differed when comparing the age groups. The highest counts were detected one week after birth, and the lowest counts were at eight months post-weaning; generally, counts were higher in pre-weaned goats compared to the post-weaned goats. Although not many studies have been published on the age-related dynamics of LAB specifically, in animals, our findings agree with studies that showed LAB, especially *Lactobacillus*, decreased with increased age in pigs [44] and humans [45]. Our results also agree with a number of metagenomic-based studies that have evaluated gut microbiota diversity in goats, including age- and health-status-related dynamics [14,15,16,17]. In two of the studies of pre-weaned goats, temporal and spatial differences in Lactobacillus abundance were detected [14] as well as a decrease in Lactobacillus in the rumen, as the goats transitioned from a milk-fed diet [16]. In this study, a diverse species of lactic acid bacteria was detected in all age groups, including those belonging to *Lactobacillus*, *Pediocococcus*, *Enterococcus* and *Weisella* spp. Two dominant LAB species, *L. reuteri* and *Pediococcus* spp., were detected in goats in this study. The *Lactobacillus* species included mostly *L. reuteri*, *L. fermentum*, *L. johnsonni*, *L. murinus*, *L. plantarum*, *L. salivarius*, *L. brevis*, *L. oris, L. amyolovorus/acidophilus*, *L. agilis*, *L. mucosae*, *L. animalis*, *L. saemneri* and *W.-confusa*. While not many studies have reported on isolation-based LAB data on the diversity and abundance from goats, the results are parallel to those reported in a recent study on lactic acid bacteria diversity in young piglets. In the latter study, the structure and predominant lactic acid bacteria changed significantly between birth and weaning [46].The current study findings also agree with the few studies that have detected *Lactobacillus* species as common inhabitants in the gut of ruminants including goats [47] and calves [48]. In the latter study involving young calves, *L. johnsonni*, *L. salivarius*, *L. murinus*, *L. mucosae*, *L. amyolovorus* and *Enterococcus* spp. were detected, but the other *Lactobacillus* species were not. In the study on goats, *L. reuteri* was the main *Lactobacillus* investigated. Unlike this study, the former studies did not evaluate age-based differences in the prevalence or diversity of LAB. Similar to the species isolated in this study, LAB were also isolated from adult cattle feces previously, including *L. acidophilus*, *L. fermentum*, *L. salivarius*, *L. brevis* and *P. acidilactici* [49]. In another study evaluating fecal samples from dairy cows, the most common lactic acid bacteria detected were *L. gasseri*, *L. reuteri*, and *L. salivarius* [50]. In this study, we report previously unreported findings on changes in abundance and also age-based differences in the diversity of LAB in goats. Although the study is cohort-based, the age-based differences in LAB abundance and diversity have broader applications in informing the future development and selection of probiotic strains for use in animals. These findings call for more research targeting other animal hosts and different production systems to evaluate if similar age-specific adaptations exist for certain species of LAB.

*Lactobacillus reuteri* was the most frequently detected species in pre-weaned and peri-weaned goats in this study. Interestingly, one month after weaning, this species was rarely detected. To our knowledge, no other study has reported these age-based changes in the predominant LAB in goats. *L. reuteri* is known to colonize the gastrointestinal tract of both human and animals with many gut health benefits attributed to its presence in the host [51]. Based on the RAPD genotyping of goat isolates in this study, a high diversity of *L. reuteri* strains were detected. In some cases, these genotypes were associated with certain age groups, while others were detected across the age groups. Some genotypes were more frequently detected, while others were rare. Further testing will reveal if these genotypes differ in the biochemical and desirable probiotic characteristics. In studies conducted in humans, *L. reuteri* isolated from the reproductive tract of women also differed in their RAPD genotypes [52]. In previous studies, LAB of the same species isolated from same host were found to differ in their beneficial characteristics [48,53]. Genome analysis of *L. reuteri* isolated from goats in Mongolia indicated differences compared to isolates from cows, sheep and horse [47]. *L. reuteri* were also the most frequently detected LAB in pigs [41,54] including pre-weaned pigs [46]. The beneficial effects of *L. reuteri* include the production of antimicrobial molecules (organic acids, ethanol and reuterin) [55] and immunomodulatory effects [51]. In addition, the most promising LAB strains with excellent probiotic potentials from broilers identified with API and 16S rRNA sequencing included *L. reuteri* among others [56]. The role of *L. reuteri* in maintaining the normal function of the digestive system and its impact on the health and wellbeing in goats, including pre-weaned goat kids, is still not clear and should be further studied. Future studies will seek to further evaluate potential benefits of these diverse genotypes in goats. *L. johnsonni* and *L. fermentum* were also detected in the goats in this study, albeit in a lesser number of goat kids than *L. reuteri*. *L. johnsonni* were detected in both pre-weaned and post-weaned goats, while *L. fermentum* were only isolated in pre-weaned goats. This may point to the adaptation of the two strains to different diets available to the goats at different ages. Interestingly, *L. johnsonni* genotypes detected in pre-weaned animals were different from those detected in post-weaned animals. This again points to a possible difference in an ecological adaptation of the strains in different age groups. *L. johnsonnii* strains have been detected in goat milk [57,58]. Benefits of this strain in ruminants have also been reported [59].The beneficial effect of *L. fermentum* strains, including antimicrobial, immune enhancement, cholesterol lowering and antioxidative properties, have been described in many studies [60,61,62,63,64]. Unlike our study where *L. fermentum* was predominantly detected in pre-weaned goats, *L. fermentum* were isolated in the rumen of a one-year-old goat in a previous study [65]. This may be due to the source of samples (rumen) versus fecal samples and the abundance of LAB in the different niches in the gut of goats or the type of feed available to the animals. *L. fermentum* are commonly isolated in dairy products from goats [66,67], which may explain why it was more common in goat kids during the pre-weaning period in this study. They are also commonly isolated from the gut of poultry and swine [64]. Studies, however, documenting the presence of these strains in the gut of goats are rare, thus this study further adds onto the understanding of the diversity, establishment and persistence of these strains in goats. Other LAB, including *L. mucosae* reported in in this study, were in older goats, especially post-weaned goats. Some studies using the species isolates from sheep, donkey milk and goat cheese found beneficial characteristics in mice [68] and in vitro studies [69,70]. There are no studies reporting potential isolation or benefits of *L. mucosae* from a goat’s gastrointestinal tract. Similarly, there are no published studies on the presence or prevalence of the rarely detected *Lactobacillus* spp. reported in this study of the gut of goats, including, *L. murinus*, *L. plantarum*, *L. salivarius*, *L. brevis*, *L. oris*, *L. amyolovorus/acidophilus*, *L. animalis*, *L. agilis* and *W. confusa*. However, these strains have been described as having beneficial characteristics in many studies in human and animals and may potentially confer similar benefits in goats [71,72]. *Lactobacillus salivarius* was the second-largest group found in a study on pigs [41] and the third group in cattle [49]. Thus, further exploration of the potential benefit of these isolates from goats may reveal those with the potential to develop as goat probiotics. The *Pediococcus* spp. group was the second-largest group in this study of the gut of pre-weaned goats. They were detected rarely in goats one month after weaning. *Pediococcus* spp. are commonly detected in food products, especially in fresh and fermented dairy from goats [73,74,75]. A recent study reported *Pediococcus* spp. in silage, some strains of which showed antimicrobial effects against pathogenic bacteria [76]. *Pediococcus* spp. Were also detected in rumen liquor from goats in one study [77]. The potential beneficial attributes of *Pediococcus* strains have been highlighted in some studies in humans, animals and fish, in vitro [75,77,78,79,80]. A review of the beneficial attributes of the *Pediococcus* spp. has also been published recently [81]. Thus, although not many studies have reported their isolation in the gut of goats or on the effects in the goats, there is potential that some strains may produce beneficial compounds that need further exploration. The *Enterococcus* species was detected in fecal samples from goats four months and older in this study and none in younger goats. Enterococcus are known to be common inhabitants in the gut of animals [82] but have also been detected in goat and sheep colostrum and milk in previous studies [83,84]. However, studies reporting on their diversity and age-based differences in abundance in the gut of goats are rare.

The significance of the intra-species RAPD genotypic diversity revealed in this study remains to be evaluated. The highest diversity was detected in *L. reuteri*, but other species including *L. johnsonni*, *L. plantarum* and *L. fermentum* displayed intra-species diversity. This is especially important since it was also associated with the age of goats at which the strains were found. Since the bacterial genome content ultimately confers the host with ability to establish in a niche, it is possible this was the case with the different genotypes of the same species reported in goats. With further characterization, the functional and metabolic significance of the intra-species genotypic diversity may be evident.

## 5. Conclusions

This study has described age-based dynamics in the abundance, richness and diversity of LAB in growing pastured goats using a one-year study of a cohort. The generated information is novel and points to potential age-based differences that are important considerations in the future development of host- and age-specific probiotic species for goats, and potentially other animals, with further research. Future research targeting other goat production systems and other animal species will reveal if similar LAB dynamics exist in resident hosts.

## Figures and Tables

**Table 1:** Diversity and prevalence of lactic acid bacteria isolated from fecal samples of growing pastured goats at different ages (number of animals).

LAB spp.	2 Days (35)	7 Days (28)	14 Days (28)	28 Days (31)	56 Days (34)	0 DPW (28)	2 DPW (32)	7 DPW (31)	1 MPW (32)	4 MPW (30)	8 MPW (30)
*L. reuteri*	30	15	21	21	34	25	27	18	-	-	3
*L. fermentum*	9	4	7	3	3	-	-	-	-	-	-
*L. johnsonni*	7	6	2	3	5	3	1	1	3	-	6
*L. murinus*	3	-	4	1	-	-	-	2	-	-	-
*L. salivarius*	-	1	-	1	-	-	1	3	-	-	-
*L. plantarum*	1	1	2	1	-	-	1	-	-	10	2
*L. brevis*	-	1	-	-	-	-	-	-	-	-	1
*L. oris*	-	-	-	1	-	-	-	-	-	-	-
*L. acidophilus/amyolovorus*	-	-	-	-	1	-	2	1	-	-	-
*Pediococcus* spp.	26	16	22	20	15	2	3	9	8	3	1
*L. mucosae*	-	-	-	-	-	12	14	25	32	29	28
*L. animalis*	-	-	-	-	-	-	-	-	-	1	10
*Enterococcus* spp.	-	-	-	-	-	-	-	-	-	6	12
*L. agilis*	-	-	-	-	-	2	-	2	-	-	-
*Weisella confusa*, *W. cibaria*	-	-	-	-	-	-	-	-	-	-	2
*L. saerimneri*	-	-	-	-	-	-	1	1	-	-	1

## Data Availability

The raw data are available upon request from the corresponding author.

## Supplementary Materials

The following supporting information can be downloaded at https://www.mdpi.com/article/10.3390/ani13162669/s1, Figure S1: Example of M-13 primer generated RAPD PCR profiles of LAB isolates from goats and used to differentiate genotypes and select isolates for sequencing. 2–6 similar profiles (same genotype), 1(highly similar to 2–6 but with unique bands), 7,9,10,13,15-unique genotypes, 8,11-similar profiles (same genotypes), and 12,14-similar profiles (same genotype). L-1kb ladder (NEW ENGLAND BioLabs® Inc.); Table S1: Sequences of a 676 bp fragment of 16s rDNA of different lactic acid bacteria isolated from fecal samples of goats; Table S2: Primers used in the study.
