# Supplementary material for: Understanding Age-Related Longitudinal Dynamics in Abundance and Diversity of Dominant Culturable Gut Lactic Acid Bacteria in Pastured Goats
Source: Animals (Basel). 2023 Aug 19;13(16):2669. doi: 10.3390/ani13162669 (PMC10451344; doi:10.3390/ani13162669)
Supplement: Supplementary file 1 [file animals-13-02669-s001.zip › Suplementary table S2-Primers used in the study.pdf]

Supplementary Table S2: Primers used in the study

Primers	Primer sequence	Fragment size	Ref
qPCR primers F	5-AGCAGTAGGGAATCTTCCA-3	341	36
qPCR primer R5	5-CACCGCTACACATGGAG-3		
Sequencing primer 9f	5-GAGTTTGATCCTGGCTCAGGA-3	676	41
Sequencing primer 685r	5 -TCTACGCATTTCACCGCTAC-3		
M13 RAPD primer	5-GAGGGTGGCGGTTCT-3	-	37
