# Supplementary material for: Understanding Age-Related Longitudinal Dynamics in Abundance and Diversity of Dominant Culturable Gut Lactic Acid Bacteria in Pastured Goats
Source: Animals (Basel). 2023 Aug 19;13(16):2669. doi: 10.3390/ani13162669 (PMC10451344; doi:10.3390/ani13162669)
Supplement: Supplementary file 1 [file animals-13-02669-s001.zip › Supplemetary figure 1.pdf]

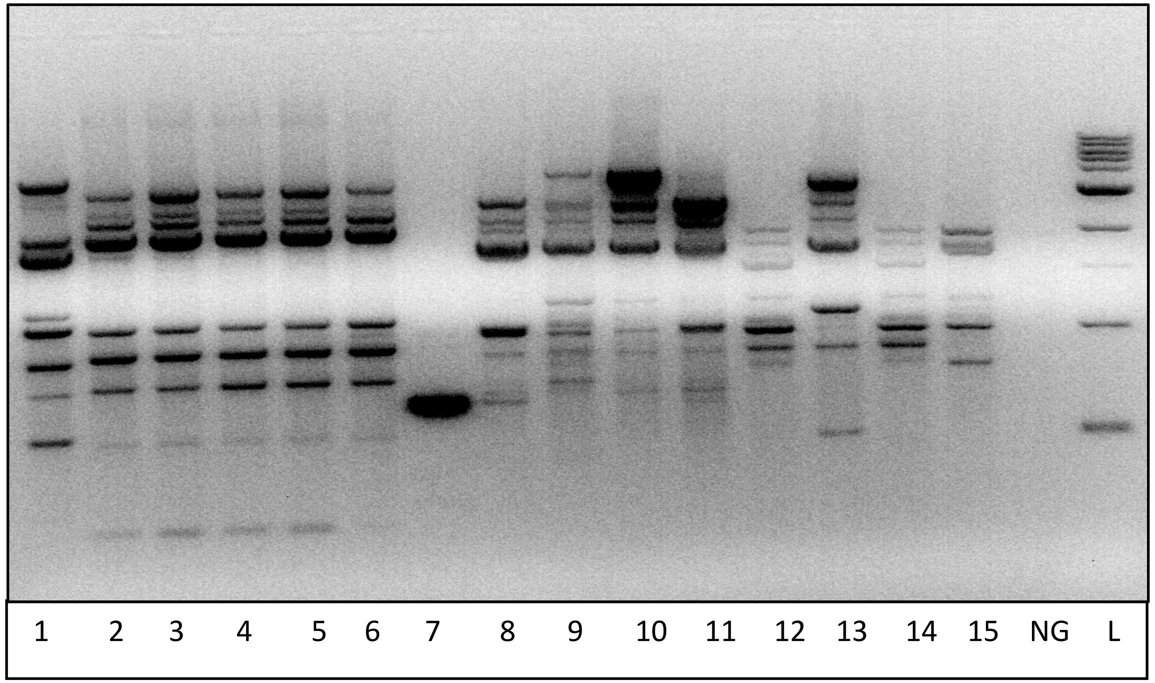

Supplementary Figure S1 : Example of M-13 primer generated RAPD PCR profiles of LAB isolates from goats and used to differentiate genotypes and select isolates for sequencing. 2-6 similar profiles (same genotype), 1(highly similar to 2-6 but with unique bands), 7,9,10,13, 15-unique genotypes, 8,11-similar profiles (same genotypes), and 12,14-similar profiles (same genotype). L-1kb ladder (*NEW ENGLAND BioLabs®* Inc).

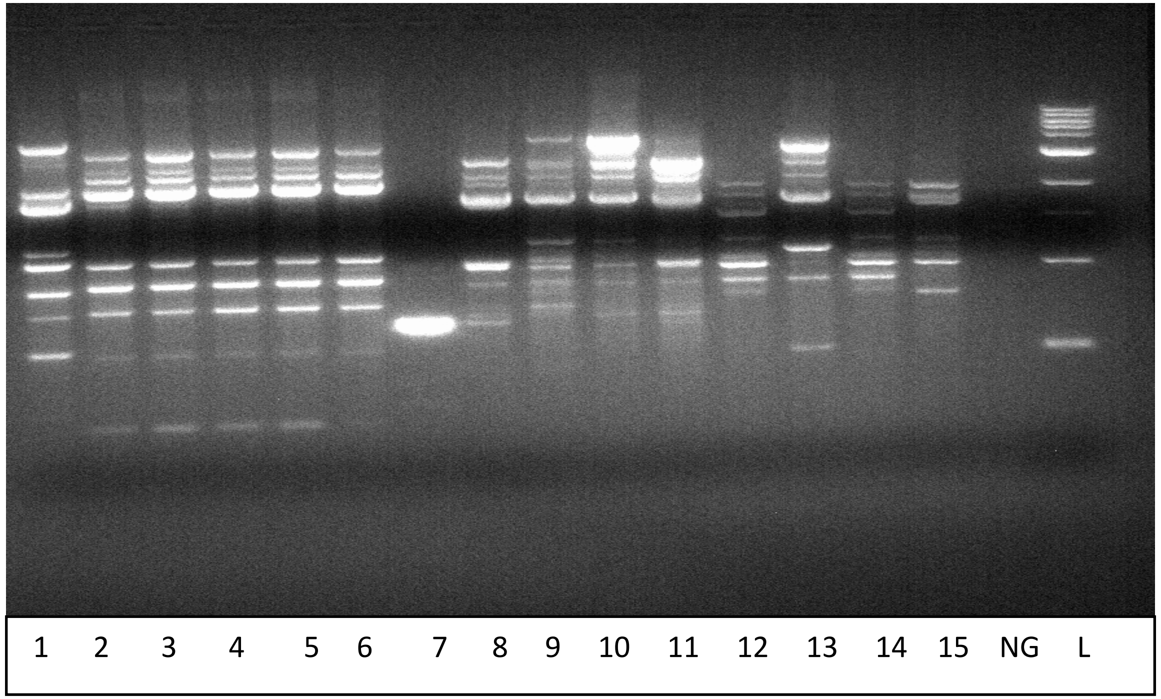

Supplementary Figure S1: Example of M-13 primer generated RAPD PCR profiles of LAB isolates from goats and used to differentiate genotypes and select isolates for sequencing. 2-6 similar profiles (same genotype), 1(highly similar to 2-6 but with unique bands), 7,9,10,13, 15-unique, 8,11-similar profiles (same genotypes), and 12,14-similar profiles (same genotype). L-1kb ladder (*NEW ENGLAND BioLabs*® Inc).
